# Microbiota regulates bone marrow mesenchymal stem cell lineage differentiation and immunomodulation

**DOI:** 10.1186/s13287-017-0670-7

**Published:** 2017-09-29

**Authors:** E Xiao, Linhai He, Qiong Wu, Junxiang Li, Yang He, Lu Zhao, Shuo Chen, Jingang An, Yansong Liu, Chider Chen, Yi Zhang

**Affiliations:** 1 Department of Oral and Maxillofacial Surgery, Peking University School and Hospital of Stomatology, #22 Zhongguancun South Avenue, Haidian District, Beijing, 100081 China; 2 National Engineering Laboratory for Digital and Material Technology of Stomatology, Beijing Key Laboratory of Digital Stomatology, #22 Zhongguancun South Avenue, Beijing, 100081 China; 3 Department of Anatomy and Cell Biology, University of Pennsylvania, School of Dental Medicine, Philadelphia, PA 19104 USA; 4 MOE Key Laboratory of Bioinformatics, Center for Synthetic and System Biology, Tsinghua University, Beijing, China; 5 School of Life Sciences, Tsinghua University, Beijing, 100084 China; 6 Chinese Academy of Sciences Shanghai Laboratory Animal Center, Songjiang District, Shanghai, 201615 China

## Abstract

Health is dependent on the homeostasis of both inner and external microenvironments. The microbiota as the external microenvironment plays a critical role in regulation of several organ systems in mammals. However, it is unclear whether the microbiota regulates homeostasis of the skeletal system and bone marrow mesenchymal stem cells (BMMSCs). Here, using a well-established germ-free (GF) mouse model, we show that the microbiota significantly alters the stemness of BMMSCs in comparison to specific-pathogen-free (SPF)-derived BMMSCs. Colonization of GF mice with SPF microbiota (conventionalized (ConvD)) normalizes the proliferation and differentiation abilities of BMMSCs. On the other hand, normal microbiota is required to maintain immunomodulatory properties of BMMSCs through induction of activated T-cell apoptosis and cytokine secretion. GF-derived BMMSCs lose the capacity to ameliorate disease phenotypes in dextran sulfate sodium-induced experimental colitis mice. Mechanistically, single-cell RNA-sequencing analysis shows that ConvD BMMSCs have a similar gene expression pattern to SPF-derived BMMSCs, which have a distinct gene distribution from GF-derived BMMSCs.

**Electronic supplementary material:**

The online version of this article (doi:10.1186/s13287-017-0670-7) contains supplementary material, which is available to authorized users.

## Background

The mammal is inhabited by a vast number of bacteria, archaea, viruses, and eukaryotes. This microorganism coexistence with their hosts is referred to as the microbiota. It is reported that the human microbiota contains as many as 10^14^ bacterial cells, a number 10 times greater than the number of human cells [1]. The microbiota colonizes on the host mammal after they are exposed to the external environment. More than a billion years of mammalian–microbial coevolution has led to interdependency, resulting in a critical role of the microbiota in hematopoiesis [2], immune system development [3], neurologic signaling [4], host metabolism [5], and bone mass remodeling [6].

Bone marrow mesenchymal stem cells (BMMSCs), a kind of adult stromal cell in bone marrow, both contribute to the bone turnover [7] and form the unique bone marrow niche with hematopoietic stem cells [8]. BMMSCs show promising therapeutic potential based on their multipotent differentiation potential and immunomodulatory capacity [9, 10]. However, whether these mesenchymal stem cells are “born with” these fantastic capacities or are educated by the microbiota was still not known. Thus, in this study we aimed to elucidate the effect of the microbiota on the multipotent differentiation and immunomodulatory abilities of BMMSCs.

## Results and discussion

### BMMSCs from germ-free mice exhibited higher colony forming ability and proliferation rate

**Fig. 1:**
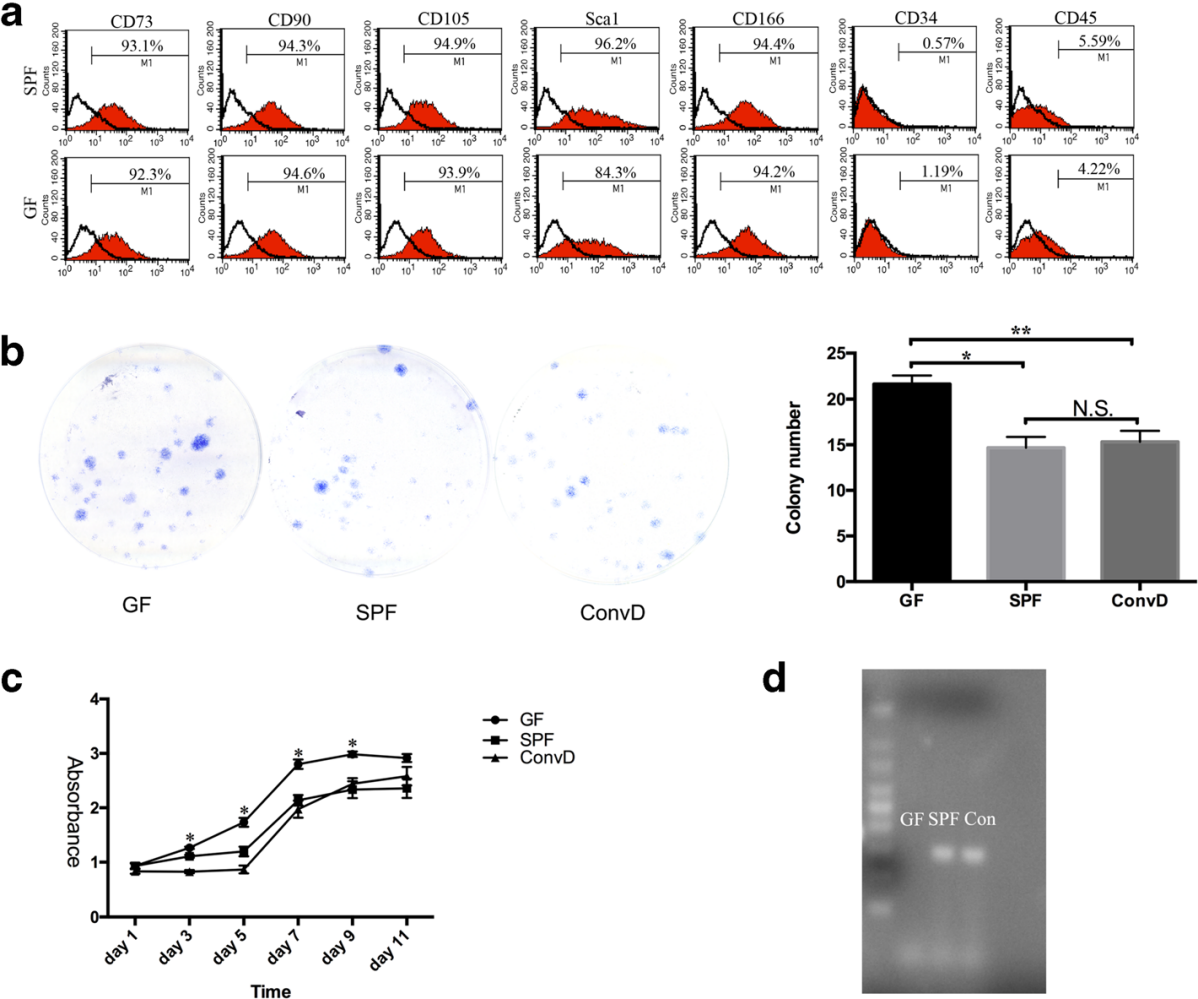
Characterization and proliferation of GF, SPF, and ConvD BMMSCs. **a** Flow cytometry analysis shows that BMMSCs derived from SPF and GF mice had similar positive markers (CD73, CD90, CD105, Sca1, and CD166) and negative markers (CD34 and CD45). **b** Colony forming unit experiments show BMMSCs derived from GF mice had higher colony forming rate compared to that from SPF and ConvD mice (21.67 ± 0.88 vs 14.67 ± 1.20 and 15.33 ± 1.2). **c** CCK8 analysis shows BMMSCs from GF mice had higher proliferation capacity compared to that from SPF and ConvD mice. **d** PCR analysis shows that no bacterium was detected in GF feces using universal bacteria primers 515F and 806R. All experimental data verified in at least three independent experiments. Error bars represent the SEM from the mean values. **P < 0.001; *P < 0.05. ConvD conventionalized, GF germ free, SPF specific pathogen free, N.S. not significant

To examine whether BMMSCs are regulated by the microbiota, BMMSCs were isolated from germ-free (GF) and specific-pathogen-free (SPF) mice. PCR analysis showed no bacteria detected in GF feces by universal bacteria primers, while conventionalized feces from GF mice colonized with SPF microbiota showed a similar pattern to the SPF group (Fig. 1d). Flow cytometry analysis showed that these two cell groups expressed similar mesenchymal cell surface markers CD73, CD90, CD105, CD166, and Sca1, while being negative for hematopoietic cell surface markers CD34 and CD45 (Fig. 1a). To examine the colony forming ability, 1 million BMMSCs were seeded in 60-mm dishes to test the colony forming unit rate. Our data indicated that BMMSCs from GF mice formed significantly more colonies compared with those from the SPF mice (Fig. 1b). To further confirm the effects of the microbiota in BMMSC colony forming ability, GF mice were exposed in a conventional environment by cohousing with SPF mice for 2 weeks (conventionalized (ConvD)). The colony forming ability was significantly decreased to the level of SPF mice-derived BMMSCs (Fig. 1b). Next, using cell count kit 8 (CCK8), BMMSCs derived from GF mice also showed a higher proliferation rate when compared to SPF and ConvD BMMSCs (Fig. 1c). Besides, cell cycle analysis also showed more G2 and S-phase cell percentage in GF-derived BMMSCs compared with that of SPF, and showed less Annexin V-positive cells in GF-derived BMMSCs (Additional file 1: Figure S1D, E). Taken together, these data elucidate that the microbiota functionally controls BMMSC self-renewal capacities.

### Microbiota increases adipogenesis but decreases osteogenesis of BMMSCs

**Fig. 2:**
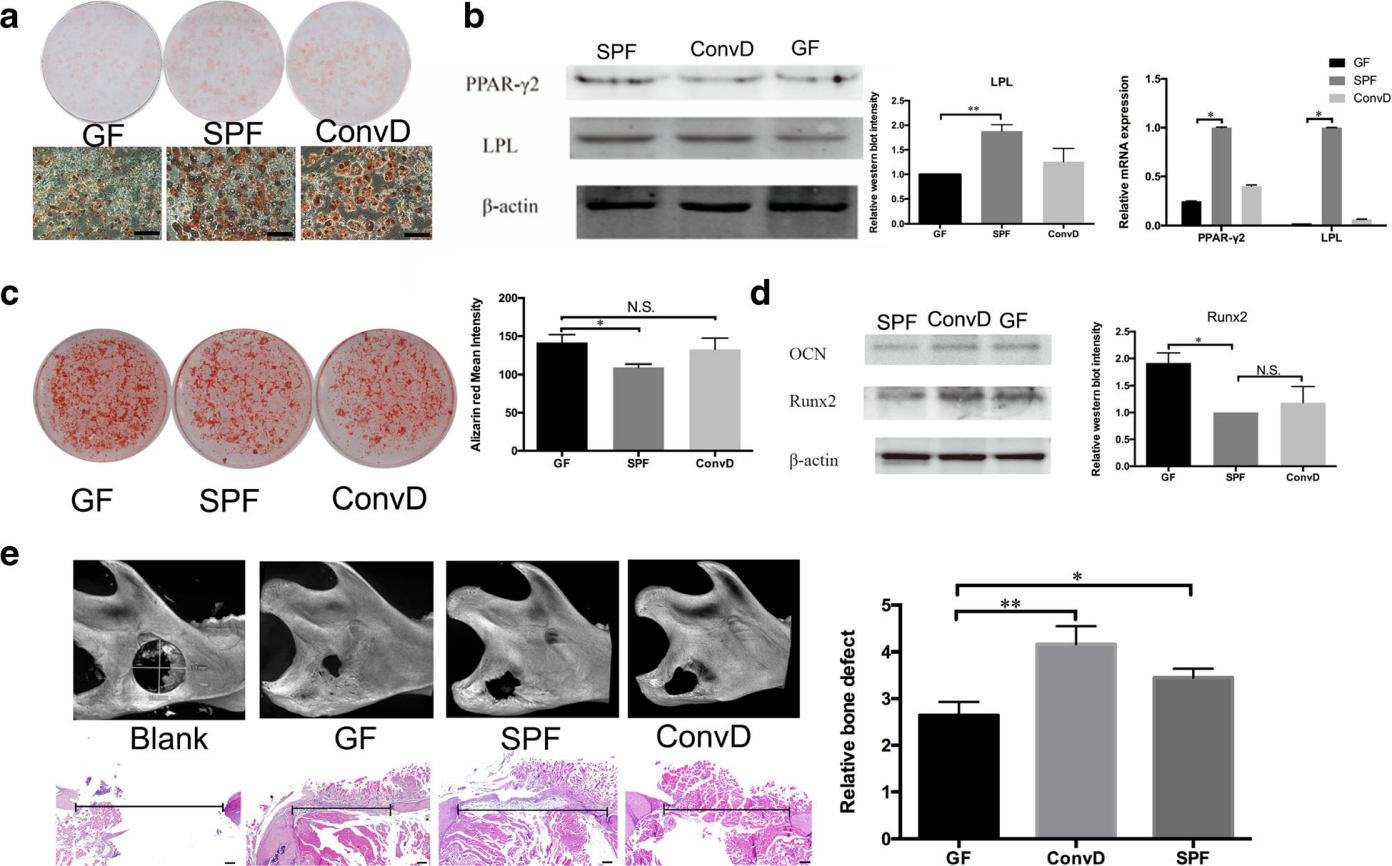
Microbiota governs BMMSC lineage commitment. **a** Oil Red O staining. Left panel: BMMSCs from GF mice show less Oil Red O-positive adipocytes after adipogenic induction in vitro compared to BMMSCs derived from SPF (middle panel) and ConvD (right panel) mice (bar = 100 μm). **b** Western blot analysis shows that SPF-derived and ConvD-derived BMMSCs express higher adipogenic key transcription factor PPAR-γ and adipogenic marker LPL compared to germ-free BMMSCs (left and middle). Real-time PCR shows PPAR-γ and LPL mRNA highly expressed in germ-free derived BMMSCs (right). **c** Alizarin Red staining shows GF-derived BMMSCs formed more mineralized nodule in vitro compared to SPF and ConvD groups. **d** Western blot analysis shows that GF BMMSCs expressed higher OCN and Runx2 after osteogenic induction in vitro (left). Quantification of Runx2 western blot intensity (right). **e** In-vivo bone regeneration capacity of BMMSCs. Left panels of micro-CT and H&E staining show the initial bone defect size in the rat mandibles. Second panels of micro-CT and H&E staining show the bone defect size after GF BMMSC treatment. Third panels of micro-CT and H&E staining show bone defect size after SPF BMMSC treatment. Right panels show the bone defect size after ConvD BMMSC treatment (bar = 200 μm). All experimental data verified in at least three independent experiments. Error bars represent the SEM from the mean values. **P < 0.001; *P < 0.05. ConvD conventionalized, GF germ free, SPF specific pathogen free, N.S. not significant, PPAR peroxisome proliferator-activated receptor, LPL lipoprotein lipase, Runx2 runt-related transcription factor 2, OCN osteocalcin

Currently, there is broad intense interest in understanding the contribution of the microbiota to vertebrate/mammalian organ systems. Germ-free mice and antibiotic-treated mice have been shown to increase bone mineral density with reduction of osteoclasts and bone resorption [6, 11]. As bone metabolism couples bone resorption with bone formation to maintain skeletal homeostasis, how the microbiota influences bone-forming cells, especially BMMSCs, is still largely unknown. To further examine the effect of the microbiota on BMMSC lineage commitment, we conducted adipogenic and osteogenic induction in vitro. Oil Red O staining showed that SPF-derived and ConvD-derived BMMSCs formed more Oil Red O-positive adipocytes than GF-derived BMMSCs (Fig. 2a). Western blot and real-time PCR analysis further confirmed that the key adipogenic transcription factors peroxisome proliferator-activated receptor γ (PPAR-γ2) and adipogenic marker lipoprotein lipase (LPL) were highly expressed in the BMMSCs from the SPF and ConvD groups after adipogenic induction, while GF-derived BMMSCs expressed significantly lower PPARγ and LPL (Fig. 2b). On the other hand, osteogenic study showed that BMMSCs from GF mice formed more mineralization deposit than SPF/ConvD BMMSCs, which was detected by Alizarin Red staining (Fig. 2c). Western blot analysis indicated that the expression levels of osteogenic transcription factor runt-related transcription factor 2 (Runx2) and osteogenic marker osteocalcin (OCN) were significantly higher in GF-derived BMMSCs when compared with SPF/ConvD BMMSCs (Fig. 2d). These data demonstrated that the microbiota significantly altered BMMSC lineage differentiation in vitro. To test the in-vivo bone formation abilities, we implanted GF-derived, SPF-derived, and ConvD-derived BMMSCs in a mandibular bone defect model to regenerate bone tissue. Micro-CT analysis showed that GF-derived BMMSCs greatly repaired mandibular bone defect, while BMMSCs from the SPF and ConvD groups showed less bone regeneration with bigger size bone defect remaining (Fig. 2e). In addition, hematoxylin and eosin (H&E) staining also showed significantly more bone regeneration in the GF BMMSC implanted group when compared with the SPF and ConvD BMMSC implanted groups (Fig. 2e). To examine whether the superior osteogenesis ability of GF BMMSCs can contribute to bone mineral density (BMD) in vivo*,* we performed micro-CT analysis to show that femoral BMD of GF mice was significantly increased compared to SFP mice (Additional file 1: Figure S1A, B). After 2 weeks of cohousing, the ConvD femoral BMD was largely decreased to the level of SPF mice (Additional file 1: Figure S1A, B).

Collectively, these findings indicated that the microbiota inhibited BMMSC proliferation and adipogenesis but increased osteogenesis and in-vivo regenerative abilities. Interestingly, the microbiota increases adipogenic differentiation, which is consistent with reduced obesity in germ-free mice [12]. Probiotic and prebiotic treatments are able to increase bone mass and BMD, indicating that intestinal microbiota may impact bone metabolism and health maintenance [13–16]. These findings connect gut microbiota and skeletal remodeling, which prompts us to investigate the impact of the microbiota on BMMSCs and bone tissue regeneration. They also suggest that physiological regulation of the osteoblastic/adipogenic lineage switch in the bone compartment involves the microbiota.

### BMMSCs from GF mice were deficient in immunomodulation

**Fig. 3:**
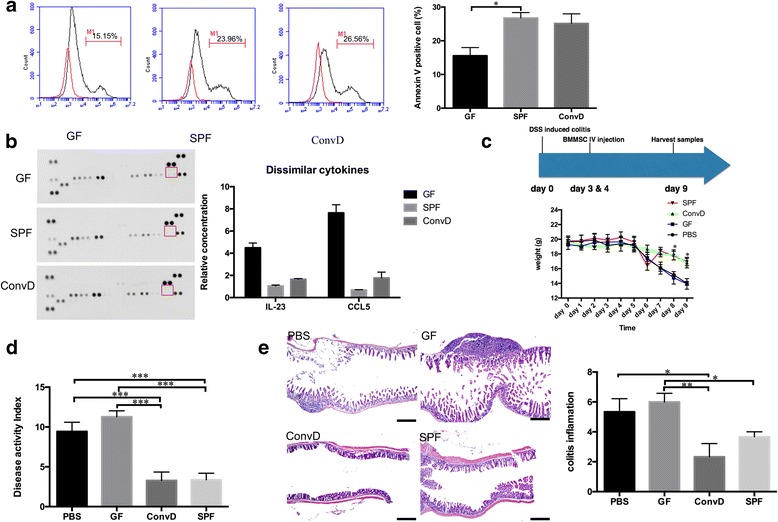
Microbiota is required for BMMSC immunomodulation. **a** Flow cytometry analysis shows that BMMSCs induced T-cell apoptosis by detecting Annexin V-positive cells in a coculture system. SPF-derived and ConvD-derived BMMSCs induced more T-cell apoptosis. **b** Cytokine array shows that GF-derived BMMSCs expressed more IL-23 and CCL5 when compared to the other two groups exposed to the microbiota. **c** Infusion of SPF and ConvD BMMSCs rescue weight loss in DSS-induced colitis mice, while GF BMMSCs had comparable weight loss to the PBS control. **d** Disease activity index shows that BMMSCs from GF mice did not decrease the disease activity, while the other two groups of BMMSCs decreased the disease activity index. **e** PBS and GF BMMSC treated colitis mice show severe epithelial disruption and inflammatory cell infiltration compared to ConvD and SPF BMMSC treated colitis mice (bar = 500 μm). All experimental data verified in at least three independent experiments. Error bars represent the SEM from the mean values. ***P < 0.005; **P < 0.001; *P < 0.05. BMMSC bone marrow mesenchymal stem cell, CCL5 chemokine C–C motif ligand 5, ConvD conventionalized, DSS 3% dextran sulfate sodium, GF germ free, IL interleukin, PBS phosphate-buffered saline, SPF specific pathogen free

Next, we asked whether the microbiota can affect BMMSC immunomodulation. It has been reported that BMMSCs cannot suppress immune reactions, unless they were preactivated by certain combinations of the inflammatory environment [17, 18]. The microbiota played a critical role in the maturation of immune system and tolerance [19, 20]. Thus, we reasoned that the immunomodulatory capacities of BMMSCs may be affected by the microbiota. We first cocultured BMMSCs from different groups with pan-T cells and examined their abilities to induce T-cell apoptosis. Flow cytometry analysis showed that SPF-derived and ConvD-derived BMMSCs significantly induced more T-cell apoptosis when compared with BMMSCs from germ-free mice (Fig. 3a). In addition, cytokine array analysis showed that GF-derived BMMSCs secreted increased proinflammatory factors interleukin-23 (IL-23) and chemokine C–C motif ligand 5 (CCL5) (Fig. 3b) which contributed to inflammatory disease. To further investigate the ability for immunomodulation of BMMSCs, the GF, SPF, and ConvD BMMSCs were systemically infused into a 3% dextran sulfate sodium (DSS)-induced experimental colitis mouse model [21] at 3 days post DSS induction (Fig. 3c). The colitis mice had significantly reduced body weight compared to C57BL6 control mice from days 5 to 9 post DSS induction (Fig. 3c). After infusion of either SPF-derived or ConvD-derived BMMSCs, but not GF BMMSCs, the body weight of the colitis mice was partially rescued at 9 days post DSS induction (Fig. 3c). The disease activity index (DAI), including body weight loss, diarrhea, and bleeding, was significantly elevated in the colitis mice compared to the control group. After infusion of SPF and ConvD BMMSCs, the DAI in the colitis mice was obviously decreased at day 9, while GF BMMSC infusion failed to reduce the DAI (Fig. 3d). BMMSCs from microorganism-exposed mice such as SPF and ConvD significantly relieved the disease activity more than twofold compared to the GF BMMSC and PBS treated groups (3.36 ± 0.48, 3.28 ± 0.61 vs 11.29 ± 0.43, 9.43 ± 0.67; Fig. 3d). In addition, H&E staining and the quantitative inflammatory score showed that the SPF-derived and ConvD-derived BMMSCs greatly decreased the colitis inflammation (inflammation scores 3.67 ± 0.33 and 2.33 ± 0.88) compared with PBS and GF BMMSC treated groups (inflammation scores 6.0 ± 0.58 and 5.33 ± 0.88; Fig. 3e).

The role of the microbiota in regulating immune cell polarization and human diseases is receiving increasing attention [22, 23]. BMMSCs reside in the skeleton to maintain osteoblastic lineage cell function and serve as a niche for hematopoietic stem cells, which involves several physiological regulations and interplays with the immune system. Our data indicated that in the unique niche of both mesenchymal stem cells and hematopoietic stem cells, the microbiota also regulated the BMMSCs, which may be related to the maturation or apoptosis of hematopoietic cell lines.

### Single-cell RNA-sequencing analysis identified three pathway categories regulated by microbiota

**Fig. 4:**
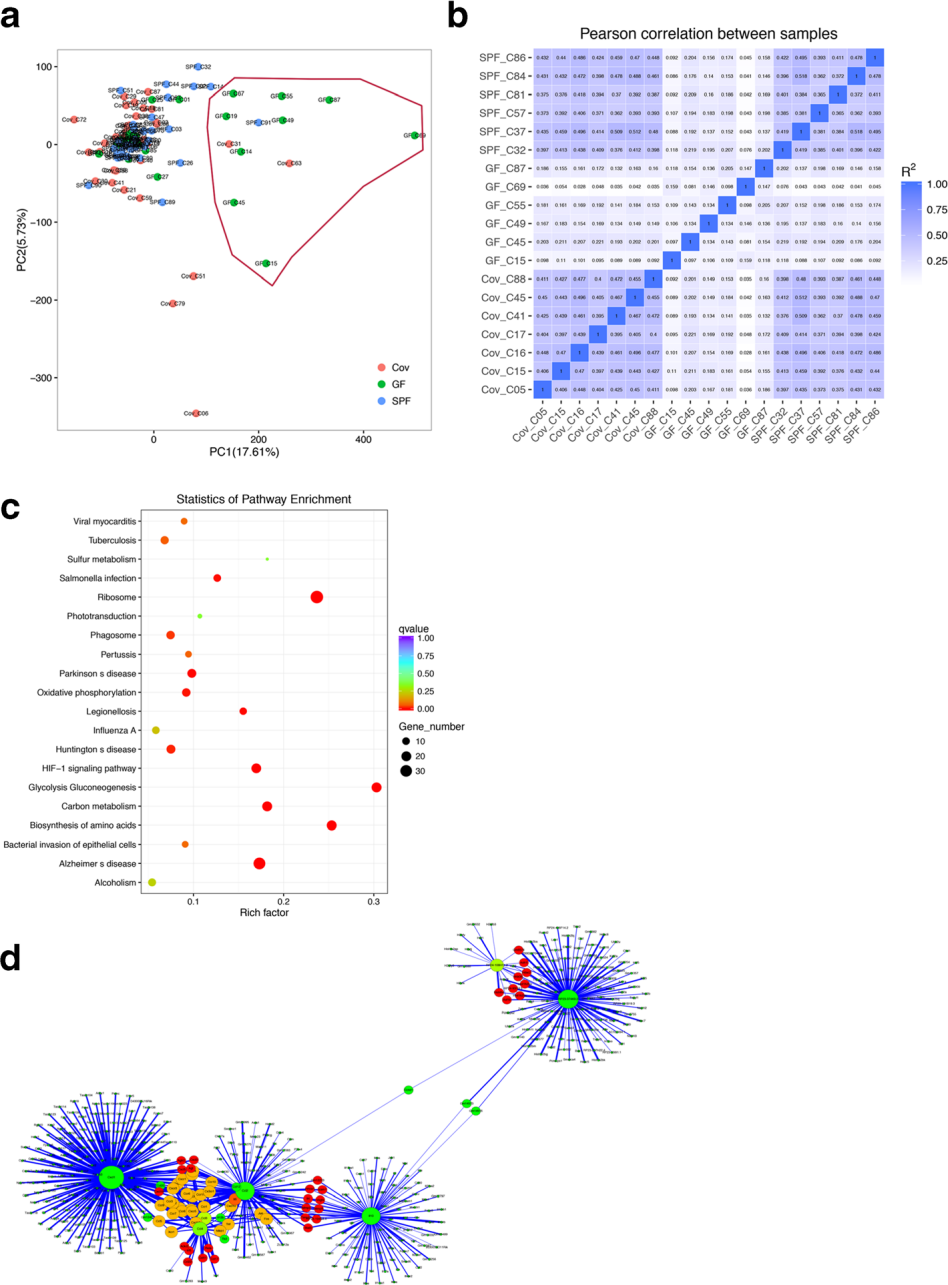
Single-cell RNA-sequencing identifies differences of three major pathway categories between GF and SPF/ConvD BMMSCs. **a** Unsupervised cell population clustering shows SPF and ConvD BMMSCs clustered together, while GF BMMSCs distributed into two parts. **b** Correlation analysis shows most mRNA expression in SPF and ConvD BMMSCs was related, distinct from GF BMMSCs. **c** Pathway analysis shows the different expressing genes between GF and SPF/ConvD BMMSCs. **d** Different gene-related protein interaction networks show that chemokines such as CXCL1, CCL2, and IL10 play a central role in BMMSC immunomodulation. GF germ free, SPF specific pathogen free

To investigate and compare the gene expression differences between GF, SPF, and ConvD BMMSCs, 35 single ConvD-derived BMMSCs, 28 single GF BMMSCs, and 32 SPF BMMSCs were isolated and sequenced. Principal component analysis showed that almost all SPF group cells cluster together and most of the ConvD group cells distributed together and closed to the SPF group cells (Fig. 4a). BMMSCs from GF mice were separated into two parts, in which 19 germ-free BMMSCs were clustered with SPF and GF group cells, while nine GF BMMSCs showed a dramatically different expression pattern (Fig. 4a). These subcluster populations may cause the different behaviors of GF BMMSCs in both self-renewal and lineage differentiation. Correlation analysis further confirmed the higher correlation between most of the SPF-derived and ConvD-derived BMMSCs (Fig. 4b), indicating that the microbiota homogenized the gene expression of BMMSCs in SPF and ConvD mice. In contrast, BMMSCs from germ-free mice contained subpopulation cells that kept the distinct expression pattern when compared with microbiota-educated BMMSCs. Single-cell RNA-sequencing data showed that there were totally 189 dissimilar gene expression between germ-free BMMSC subpopulations and microbiota-educated cells (Fig. 4b). These differentially expressed genes belonged to several pathways (Fig. 4c), and could be concluded in three major categories, including cell metabolic pathways, HIF-1/inflammatory signaling, and neurodegenerative pathways (Additional file 2: Table S1). Metabolic pathways, such as ribosome, glycolysis, amino acid biosynthesis, carbon metabolism, and oxidative phosphorylation, have been shown to play an important role in regulating BMMSC proliferation and differentiation [24–26]. In addition, HIF-1 and several infection/inflammatory signaling pathways may be involved in cytokine and chemokine secretion of BMMSCs, which is critical for BMMSC immunomodulation. Remarkably, in the different gene interaction map, microbiota-educated BMMSCs showed significantly elevated chemokines and interleukin-10 (IL-10) expression, which was also related to the capacities of immunomodulation in BMMSCs (Fig. 4d).

The microbiota is involved in the regulation of multiple host metabolic pathways, which activates immune-inflammatory axes and signaling pathways [27]. As the bone compartment is unlikely to directly contact with microbes, it is easy to envision that the microbiome could influence BMMSCs through regulating metabolic pathways. Our single-cell RNA-sequencing data further confirm that several major metabolic pathways are significantly different between GF and SPF/ConvD BMMSCs, implying metabolism may connect the microbiota and BMMSCs to maintain bone homeostasis. Besides, our results show that HIF-1 signaling may be the major regulator in BMMSC immunomodulation, since HIF-1 has been reported to crosstalk with inflammatory transcription factor NFκB and regulates release of cytokines and chemokines to control immune response [28, 29]. In summary, this is the first study to link the microbiota with BMMSC function, and single-cell RNA-sequencing analysis further provides detailed pathway prediction to connect the microbiota to regulating BMMSCs and bone metabolism.

In conclusion, we have revealed that the microbiota alters the differentiation potential and enhances the immunomodulation capacity of BMMSCs. This study provides a new point of view on how BMMSCs gain their therapeutic function.

## Additional files

Additional file 1: Figure S1. Showing comparisons of bone mineral density, bone morphology parameters, cell cycle analysis, and apoptosis cell percentages between the SPF group and the GF group. (PDF 1127 kb)

Additional file 2: Table S1. Presenting pathway enrichment analysis. (DOCX 12 kb)

Additional file 3: Supplemental materials and methods used in this study. (DOCX 24 kb)
