# Supplementary figures and images for: Microbiota regulates bone marrow mesenchymal stem cell lineage differentiation and immunomodulation
Source: Stem Cell Res Ther. 2017 Sep 29;8:213. doi: 10.1186/s13287-017-0670-7 (PMC5622543; doi:10.1186/s13287-017-0670-7)

Supplemental Figure 1

A

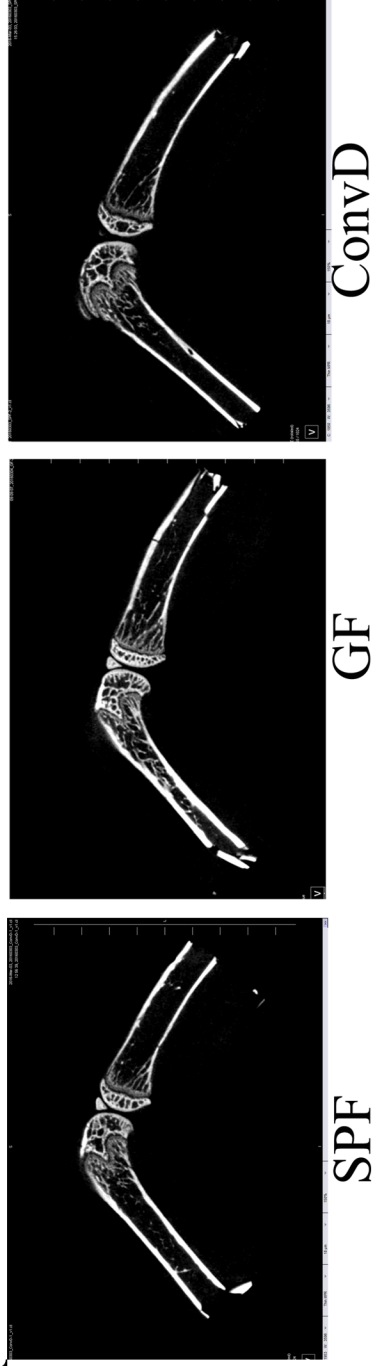

B

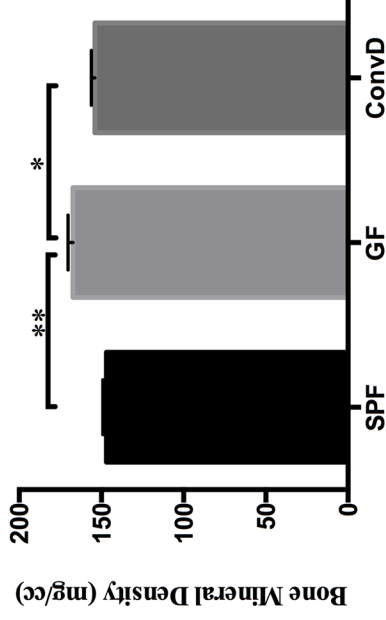

C

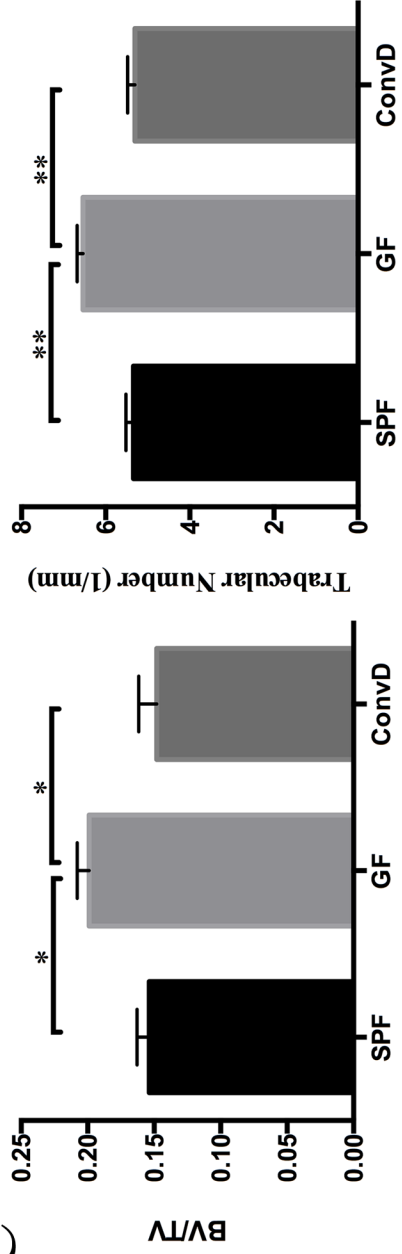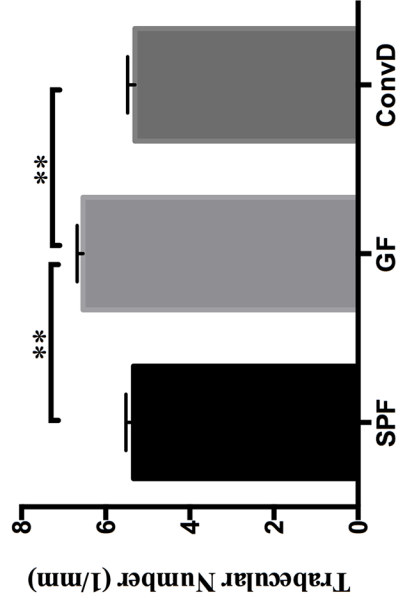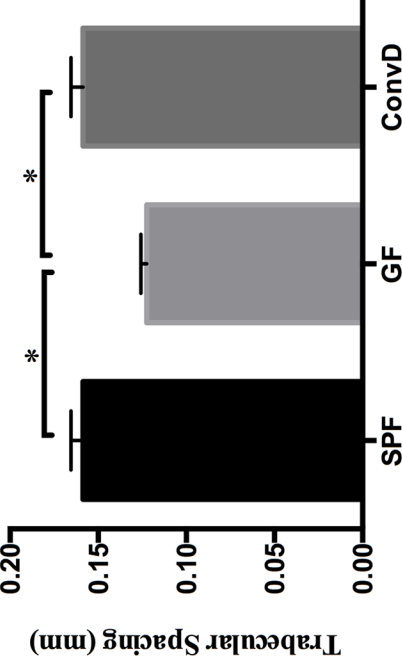

D

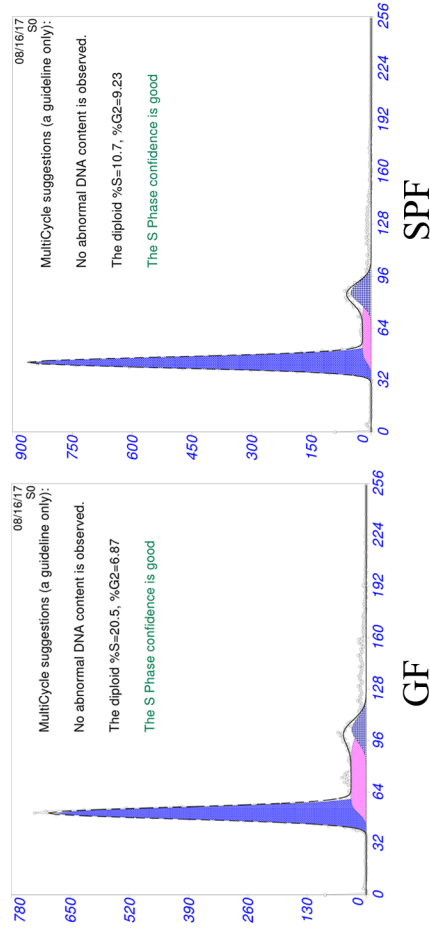

E

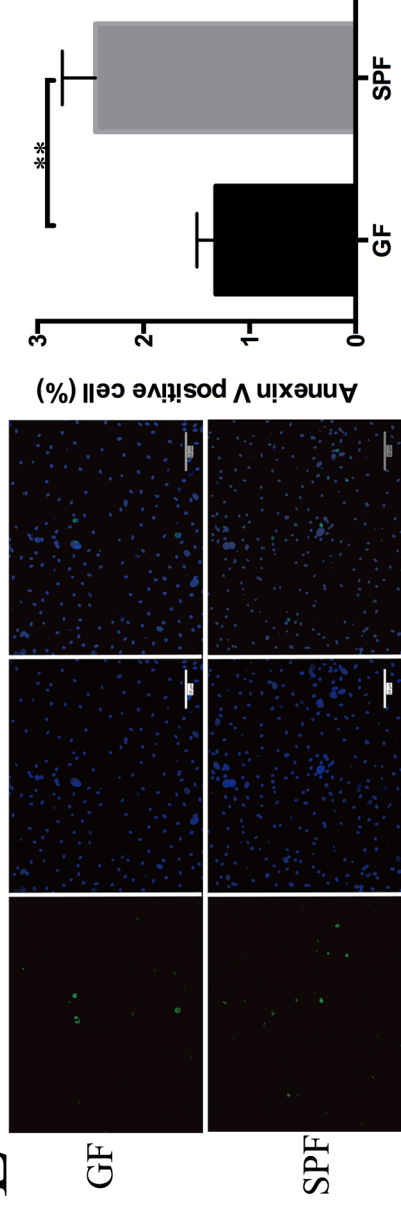

Supplement: Supplementary file 1 — Showing comparisons of bone mineral density, bone morphology parameters, cell cycle analysis, and apoptosis cell percentages between the SPF group and the GF group. (PDF 1127 kb) [file 13287_2017_670_MOESM1_ESM.pdf]
