# Supplementary material for: Microbiota regulates bone marrow mesenchymal stem cell lineage differentiation and immunomodulation
Source: Stem Cell Res Ther. 2017 Sep 29;8:213. doi: 10.1186/s13287-017-0670-7 (PMC5622543; doi:10.1186/s13287-017-0670-7)
Supplement: Supplementary file 2 — Presenting pathway enrichment analysis. (DOCX 12 kb) [file 13287_2017_670_MOESM2_ESM.docx]

**Table S1.** **Pathway enrichment analysis**

| **Pathway** | ***P* value** |
| --- | --- |
| **Category I: Metabolic pathway** |  |
| Ribosome | 1.32E-20 |
| Glycolysis / Gluconeogenesis | 9.90E-14 |
| Biosynthesis of amino acids | 1.67E-12 |
| Carbon metabolism | 3.00E-10 |
| Oxidative phosphorylation | 0.000272928 |
| **Category II: neurodegenerative diseases** |  |
| Alzheimer's disease | 1.26E-14 |
| Parkinson's disease | 5.02E-05 |
| Huntington's disease | 0.00074037 |
| **Category III: Inflammatory signaling** |  |
| HIF-1 signaling pathway | 2.31E-09 |
| Legionellosis | 7.27E-05 |
| Salmonella infection | 0.000136422 |
| Phagosome | 0.001681209 |
| Tuberculosis | 0.004965282 |
| Pertussis | 0.006064293 |
| Bacterial invasion of epithelial cells | 0.007377388 |
| Viral myocarditis | 0.007858552 |
