# Supplementary material for: Microbiota regulates bone marrow mesenchymal stem cell lineage differentiation and immunomodulation
Source: Stem Cell Res Ther. 2017 Sep 29;8:213. doi: 10.1186/s13287-017-0670-7 (PMC5622543; doi:10.1186/s13287-017-0670-7)
Supplement: Supplementary file 3 — Supplemental materials and methods used in this study. (DOCX 24 kb) [file 13287_2017_670_MOESM3_ESM.docx]

**Supplemental information**

**MATERIAL AND METHODS**

**Mice.** 8-12 weeks old Female GF and SPF C57BL/6J mice were purchased and housed in Chinese Academy of Sciences Shanghai laboratory animal center (China, Shanghai, Songjiang District). All mice were housed in the same environment under specific pathogen-free conditions with cycles of 14 hours daylight and 10 hours dark. To generate conventionalized model, GF mice were exposed in conventional environment by co-housing with SPF mice for two weeks. All animal experiments were approved by The Ethics Committee of the Peking University Health Science Center (LA2016149).

**Isolation of mouse bone marrow mesenchymal stem cells.** Bone marrow cells were flushed from mouse femurs with 2% heat-inactivated fetal bovine serum (FBS) in PBS. A single-cell suspen­sion of all nuclear cells (ANCs) was obtained by passing bone marrow cells through a 70 μm cell strainer (BD Bioscience), and seeded at a density of 1×10^7^ cells per 100 mm culture dish (Corning) and incubated at 37 °C with 5% CO_2_. To eliminate the hematopoietic cell contamination, the cultures were washed with PBS twice on the second day. The attached cells were cultured for 14 days with alpha minimum essential medium (α-MEM, Invitrogen) supplemented with 20% FBS, 2 mM L-glutamine, 55 μM 2-mercaptoethanol, 100 U/ml penicillin, and 100 μg/ml streptomycin. To confirm mes­enchymal stem cell character, we used flow cytometric analysis to show that BMMSCs were positive for CD73, CD90, CD105, CD166 and Sca-1 and negative for CD34 and CD45. For colony forming assay, 1.5x10^6^ single suspension ANCs were seeded in 60 mm culture dish (Corning). After 14 days, the dishes were washed with PBS and stained with 1% toluidine blue solution with 2% paraformaldehyde (Sigma-Aldrich). The cell clusters were counted under microscopy and those with more than 50 cells were considered as colonies.

**Cell proliferation assay.**

The second-passage BMMSCs were seeded at a density of 2,000 cells per well in 96-wells plates. The cell number was assessed on days 1, 3, 5, 7, 9 and 11 by Cell Counting Kit-8 (Dojindo Laboratories, Kumamoto, Japan) according to the manufacturer’s instructions.

**Osteogenic differentiation.** BMMSCs were cultured under osteogenic conditions containing 2 mM β-glycerophosphate (Sigma-Aldrich), 100 μM L-ascorbic acid 2-phosphate (Wako), and 10 nM dexamethasone (Sigma-Aldrich) in the growth medium. After four weeks’ induction, staining was performed to detect mineralization with 1% Alizarin Red S (Sigma-Aldrich) at room temperature. The stained areas were quantified using NIH ImageJ software and shown as a percentage of the total area.

**Adipogenic differentiation.** For adipogenic induction, 500 nM [**isobutylmethylxanthine**](http://www.ncbi.nlm.nih.gov/pubmed?term=isobutylmethylxanthine&cmd=correctspelling) (Sigma-Aldrich), 60 μM indomethacin (Sigma-Aldrich), 500 nM hydrocortisone (Sigma-Aldrich), 10 μg/ml insulin (Sigma-Aldrich), and 100 nM L-ascorbic acid phosphate were added into the growth medium. After 7 days, the cells were stained with Oil Red-O (Sigma-Aldrich), and positive cells were quantified under microscopy.

**Western immunoblotting.** Cells were lysed in M-PER mammalian protein extraction reagent (Thermo) with protease and phosphatase inhibitors (Roche), and proteins were quantified using protein assays (Bio-Rad Laboratories). Proteins were separated by SDS-PAGE and transferred to a nitrocellulose membrane (Millipore). Membranes were blocked with 0.1% Tween 20 and 5% BSA for 1 h before overnight incubation with the primary antibody diluted in blocking solution. Antibodies to mouse LPL, PPARγ and OCN were purchased from Santa Cruz Biotechnology, Inc. Antibody to mouse Runx2 was purchased from Abcam. Antibody to mouse β-actin was purchased from Sigma-Aldrich. The membranes were incubated for 1 h in HRP-conjugated secondary antibody diluted at 1:10,000 in blocking solution.

**Allogenic MSC transplantation in mandibular bone defect rat.**

To test the *in vivo* bone regeneration capacity, we created 5-mm diameter full thickness bone defect in the 8 weeks old male rat. Two million BMMSCs from three groups (n=4) were loaded on the gelatin sponge and implanted into the bone defect areas. 8 weeks after the cell transplantation, the rat mandibles were harvested and analyzed by microCT and hematoxylin and eosin staining.

**Micro-CT analysis.**

Rat mandibles were harvested and fixed in 10% formalin for 24 hours and then scanned with micro-CT (Inveon, SIEMENS, German) at 60 kV, 220 uA, 500 ms exposure time, and 882 um effective pixel size. The images were analyzed with software Inveon Research Workplace (Inveon MM, SIEMENS, USA). The bone defect images were measured by image J (Version 1.48).

**Histology.**

Rat mandibles were decalcified by 20% EDTA and then stained with H&E staining. The colons of DSS-induced colitis mice were harvested, fixed in 10% formalin overnight, and then paraffin embed for H&E staining. The colitis inflammation and DAI were analyzed as previous study[21].

**Coculture of T cells with BMMSCs.** BMMSCs (0.2 x 10^6^) were seeded on a 24-well culture plate (Corning) and incubated for 24 hr. The pre-activated pan T cells (1x10^6^) were directly loaded onto BMMSCs and cocultured for 2 days. The apoptotic T cells were detected by staining with CD3 antibody, followed by detection apoptosis by using an Annexin-V Apoptosis Detection Kit I (BD PharMingen).

**Cytokine array analysis.** Culture supernatants from BMMSCs were analyzed using a Mouse Cytokine Array Panel A Array Kit (R&D Systems) according to the manufacturer’s instructions. The results were scanned and analyzed using Image J software to calculate blot intensity.

**Allogenic MSC transplantation into acute colitis mice.** Acute colitis was induced by administering 3% (w/v) DSS (molecular mass: 36,000–50,000 Da; MP Biochemicals) through drinking water for 9 days. Passage one GF, SPF and ConvD BMMSCs were intravenously infused (1.0x10^6^ cells) into the colitis mice (n = 6) at 3 days post-DSS induction. In the control group, mice received PBS (n = 6) infusion. All mice were harvested at 9 days post-DSS induction for further analysis. Induced colitis was evaluated as previously description.

**Single cell sequencing and data analysis.** The single cells were captured by using C1 system (Fluidigm). The cell size distribution was predetermined by a Countstar instrument (ALIT instruments, China) to determine the optimal IFC for the microfluidic RNA-seq chip. The cells were loaded onto the chip at a concentration of 300–500 cells/mL and imaged *via* phase-contrast and fluorescence microscopies to assess the number and viability of cells per capture site after the capture process was complete. Only single, live cells were included in the analysis. cDNA was prepared on the same chip using a SMARTer Ultra Low RNA kit for Illumina (Clontech) after capture and imaging, according to the manufacturer’s protocol. ERCC RNA spike-in Mix (Life Technologies) was added to the selected lysis reaction and processed in parallel with RNA from selected single-cell lysates. The cDNA libraries were constructed in 96-well plates using an Illumina Nextera XT DNA Sample Preparation kit according to the protocol. Libraries were quantified using an Agilent Bioanalyzer with a highsensitivity DNA chip. Pooled libraries of 125 base pairs (bp) were paired-end sequenced using an Illumina HiSeq 2500 instrument to a depth of approximately 0.6–5 million reads per single cell sample the detailed data analysis was described in the supplemental method. Raw reads were processed using the quality control tools FASTQC and the fastx-toolkit, followed by the sequence alignment programs TopHat and SAMtools with mouse genome mm10 by the default settings. The gene expression levels were quantified as reads per kilobase per million mapped reads (RPKM) aligned using TopHat. Genes with an RPKM value less than 1 were considered not expressed. Cells that did not express the housekeeping genes *Actb* (encoding β-actin) and *Gapdh* (encoding glyceraldehyde-3-phosphate dehydrogenase) were considered unhealthy and were not considered in the analysis. For each sample, such random selections were repeated 15 times with different numbers of reads (from 0.1 to 5 million). In addition, the RPKM values were calculated with the same sequence alignment and reads count pipeline, and the genes with an RPKM less than 1 were discarded. Finally, the mean number of genes (RPKM>1) was plotted with error bars calculated based on different random repeats. The Cufflinks v2.1.1 Reference Annotation Based Transcript (RABT) assembly method was used to construct and identify both known and novel transcripts from TopHat alignment results. Alternative splicing events were classified to 12 basic types by the software Asprofile v1.0. The number of AS events in each sample was estimated, separately. The Cufflinks v2.1.1 Reference Annotation Based Transcript (RABT) assembly method was used to construct and identify both known and novel transcripts from TopHat alignment results. Differential expression analysis of germ-free group and bacteria exposed groups (SPF and ConvD group) was performed using the DESeq R package (1.10.1). DESeq provide statistical routines for determining differential expression in digital gene expression data using a model based on the negative binomial distribution. The resulting *P*-values were adjusted using the Benjamini and Hochberg’s approach for controlling the false discovery rate. Genes with an adjusted *P*-value <0.05 found by DESeq were assigned as differentially expressed. Gene Ontology (GO) consists of Molecular Function (MF), Biological Process (BP), and Cellular Component (CC) three parts, so proteins or genes can through corresponding gene ID or the method of sequence annotation find the corresponding GO term that is function category or cell localization. GO enrichment analysis of differentially expressed genes was implemented by the GOseq R package. We used KOBAS software to test the statistical enrichment of differential expression genes in KEGG pathways. PPI analysis of differentially expressed genes was based on the STRING database, which known and predicted protein-protein interactions. For the species existing in the database, we constructed the networks by extract the target gene list from the database; otherwise, Blastx (v2.2.28) was used to align the target gene sequences to the selected reference protein sequences, and then the networks were built according to the known interaction of selected reference species.

**Statistics.** Comparisons between two groups were analyzed by independent two-tailed Student’s t-tests, and comparisons between more than two groups were analyzed by one-way ANOVA. *P* values less than 0.05 were considered statistically significant.

**Supplemental figure 1. Bone mineral density was increased in GF mice.** (A) micro-CT analysis showed higher bone mineral density in GF mice when compared to SPF mice, while exposed to the microbiota decreased the bone mineral density in ConvD mice. (B) Quantification of bone mineral density in three groups. (C) Bone morphology parameters including BV/TV, trabecular bone number and trabecular spacing quantification analysis were shown. All experimental data were verified in at least 3 independent experiments. (D) Cell cycle analysis by DNA content staining showed more G2 and S phase cell in GF mice BMMSCs. (E) Annexin V immunofluorescence staining showed more apoptosis cells in SPF mice BMMSCs. Error bars represent the SEM from the mean values. ***P* < 0.001; **P* < 0.05.
